# Testing Mitochondrial-Targeted Drugs in iPSC-RPE from Patients with Age-Related Macular Degeneration

**DOI:** 10.3390/ph15010062

**Published:** 2022-01-04

**Authors:** Mara C. Ebeling, Zhaohui Geng, Madilyn R. Stahl, Rebecca J. Kapphahn, Heidi Roehrich, Sandra R. Montezuma, Deborah A. Ferrington, James R. Dutton

**Affiliations:** 1 Department of Ophthalmology and Visual Neurosciences, University of Minnesota, Minneapolis, MN 55455, USA; ebeli017@umn.edu (M.C.E.); stahl154@umn.edu (M.R.S.); kapph001@umn.edu (R.J.K.); smontezu@umn.edu (S.R.M.); 2 Stem Cell Institute, University of Minnesota, Minneapolis, MN 55455, USA; gengx@umn.edu; 3 Department of Genetics, Cell Biology, and Development, University of Minnesota, Minneapolis, MN 55455, USA; 4 Histology Core for Vision Research, University of Minnesota, Minneapolis, MN 55455, USA; rohri002@umn.edu

**Keywords:** human-induced pluripotent stem cells, retinal pigment epithelium, age-related macular degeneration, personalized drug testing

## Abstract

Age-related macular degeneration (AMD) is the leading cause of blindness in the elderly. No universally effective treatments exist for atrophic or “dry” AMD, which results from loss of the retinal pigment epithelium (RPE) and photoreceptors and accounts for ≈80% of all AMD patients. Prior studies provide evidence for the involvement of mitochondrial dysfunction in AMD pathology. This study used induced pluripotent stem cell (iPSC) RPE derived from five AMD patients to test the efficacy of three drugs (AICAR (5-Aminoimidazole-4-carboxamide ribonucleotide), Metformin, trehalose) that target key processes in maintaining optimal mitochondrial function. The patient iPSC-RPE lines were used in a proof-of-concept drug screen, utilizing an analysis of RPE mitochondrial function following acute and extended drug exposure. Results show considerable variability in drug response across patient cell lines, supporting the need for a personalized medicine approach for treating AMD. Furthermore, our results demonstrate the feasibility of using iPSC-RPE from AMD patients to develop a personalized drug treatment regime and provide a roadmap for the future clinical management of AMD.

## 1. Introduction

Age-related macular degeneration (AMD) is the leading cause of blindness in the elderly, affecting approximately more than 196 million people worldwide [1]. AMD is a degenerative process that affects the macula, a small area at the center of the retina, leading to progressive, irreversible loss of central vision. As a result, AMD patients find it difficult to perform daily tasks, such as recognizing faces, reading, or driving. There are two clinically distinct forms of the disease: the neovascular form or “wet AMD”, resulting from abnormal growth of blood vessels into the retina, and the atrophic form or “dry AMD”, resulting from loss of the retinal pigment epithelium (RPE) and photoreceptors. For wet AMD, several therapeutic interventions have been successful in attenuating or reversing disease symptoms. However, for dry AMD, which accounts for ≈80% of all AMD patients, there are currently no effective treatments.

The RPE is a pigmented cell monolayer between the retina and the outer retina blood supply known as the choroid. The RPE cell layer fulfills many key functions in the eye, including phagocytosis of shed photoreceptor outer segments, transport of nutrients from the choroid to the outer retina, and the secretion of factors that are crucial for the health and structural integrity of the retina and choroid [2]. While the mechanism responsible for AMD is not completely defined, studies of RPE from human donors with AMD have reported significant mitochondrial (mt) defects, including decreased mt mass, decreased content of electron transport chain proteins, and increased mt DNA damage [3,4,5,6,7]. In RPE cultured from donors with AMD, reduced oxidative phosphorylation was observed compared to RPE from non-diseased donors [8,9]. Since the RPE obtains most of its energy from mitochondria [10], disruptions in mt function and lower energy production could cause RPE cell death. Notably, the reported mt defects in AMD RPE occur early in the disease, before vision loss occurs. Therefore, identifying compounds that preserve or restore RPE mt function is likely an effective strategy for protecting the RPE from damage and preventing disease progression.

Although there is currently no universally effective treatment for dry AMD, several therapeutic approaches against this form of AMD have been tested in clinical trials. The most well-known clinical trial is the Age-Related Eye Disease Study (AREDS), which tested the efficacy of a nutritional supplement composed of high doses of vitamins plus the minerals copper and zinc to reduce disease progression [11]. The treatment effect was modest, as only ≈25% of patients showed reduced AMD progression [12]. Similar to reports for patients in the clinical trials, in vitro studies using primary and induced pluripotent stem cell (iPSC)-RPE cultured from AMD donors have shown significant variability and heterogeneity in the responses of individual cell lines after exposure to drugs [13,14,15,16,17]. This variation may arise from multiple etiologies proposed for AMD initiation and progression in the patient population [18] and highlights that a more personalized approach is required to match the optimal intervention to the patient-specific defect that is causing their disease.

There are several advantages of using iPSC-RPE for drug testing compared with other model systems. Animal models provide an opportunity to study how drugs that disrupt specific pathways affect retinal function. However, no animal model faithfully replicates the cardinal features of AMD, such as degeneration of the macula, which is a structure found only in primates. Primary RPE cultures have revealed important information about disease mechanisms [8,9] and have been used in drug testing [13,14]. Importantly, they cannot be isolated from living individuals. RPE made from iPSCs derived from multiple somatic cell sources enables the generation of patient-specific iPSC-RPE. The potential for utilizing iPSC-derived cells for drug screening was immediately recognized when this technology was developed [19]. iPSCs can provide an almost inexhaustible supply of cells to conduct extensive screening protocols where primary cells are unavailable or cannot be cultured in sufficient quantities. iPSCs can also be generated from a clinically defined population, thereby expanding the potential for drug discovery and drug screening projects that were not previously possible.

The focus of this study is to demonstrate the use of iPSC-RPE as a platform for identifying drugs that help maintain optimal mt function. We selected three drugs (AICAR, Metformin, and trehalose) that target pathways related to energy metabolism, mt biogenesis, and elimination of damaged mitochondria. AICAR (5-Aminoimidazole-4-carboxamide ribonucleotide) is an analog of adenosine monophosphate (AMP) that stimulates AMP-activated protein kinase (AMPK) [20] and subsequently induces mt biogenesis. Metformin, a drug that has been used to treat type 2 diabetes, is also reported to activate AMPK activity [21]. The naturally occurring sugar, trehalose, activates autophagy, which is a degradative process that eliminates defective organelles, including mitochondria [22].

Using these drugs, we tested the framework for a personalized medicine approach to match potential treatments to individuals with AMD. We generated iPSC-RPE from biopsies of conjunctival cells taken from five patients diagnosed with AMD, each characterized for the severity of the disease and genotyped for two high-risk single nucleotide polymorphisms (SNPs) associated with AMD. Then, the patient iPSC-RPE lines were used in a proof-of-concept in vitro drug screen, analyzing RPE mt function following acute and extended drug exposure. Our data suggest that the patient-specific iPSC-RPE model provides a robust tool to assess mt-targeted drug therapies for AMD.

## 2. Results

### 2.1. Proposed Drug Screening Approach to Determine Patient-Specific Treatment for AMD

**Figure 1:**
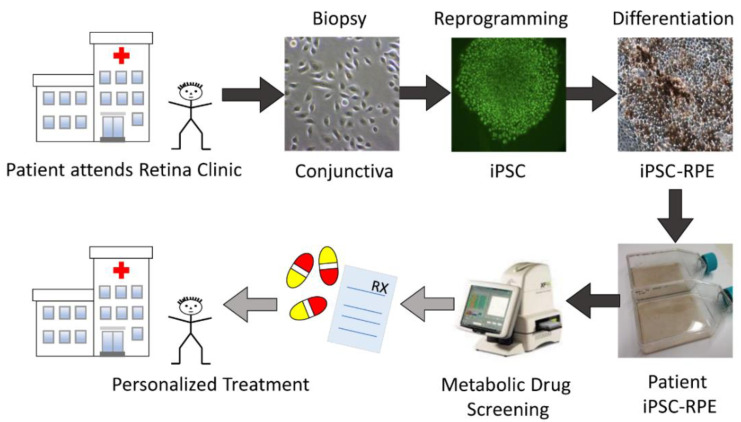
Pathway to precision medicine for patients with age-related macular degeneration. Conjunctival biopsies from patients in the AMD clinic provide somatic cells for patient-specific iPSC- line derivation and differentiation of iPSC-RPE for testing drugs to restore or protect mitochondrial function and provide clinical benefit. Dark gray arrows indicate steps included in the current study. Light gray arrows represent future steps in personalized treatment.

Figure 1 outlines the workflow required to determine the individualized response to drugs aimed at improving mt function. This process begins by obtaining somatic tissue from AMD patients, deriving patient-specific iPSC lines, differentiating iPSC-RPE, and then subsequent metabolic analysis after exposure to selected compounds. We have previously described methods for obtaining and culturing conjunctival cells for iPSC reprogramming and the generation of iPSC-RPE in studies using eyes from deceased humans (donor iPSC-RPE) obtained from the Lions Gift of Sight [23,24]. In this study, we have adapted this procedure to generate iPSC-RPE from patients with AMD (patient iPSC-RPE) for use in drug screening. Clinical translation of information from in vitro studies will assist in the development of personalized treatment regimens for each patient.

### 2.2. Characterization of iPSC-RPE Cultures Derived from AMD Patients

For this study, individual iPSC lines were generated from five patients ranging in age from 63 to 84 years. One of the patients in this study exhibited the early stages of AMD (AREDS category 2) at the time of tissue collection. Three patients scored AREDS3, indicating intermediate disease, and one was at the advanced stage of disease (AREDS4) at the time of biopsy. Four out of five patients had at least one high risk allele for Complement Factor H (CFH) (Y402H SNP), and five out of five had at least one high risk allele for age-related maculopathy susceptibility 2 (ARMS2) (A69S SNP). The demographics of the patients and the iPSC lines generated for this study are shown in Table 1.

**Figure 2:**
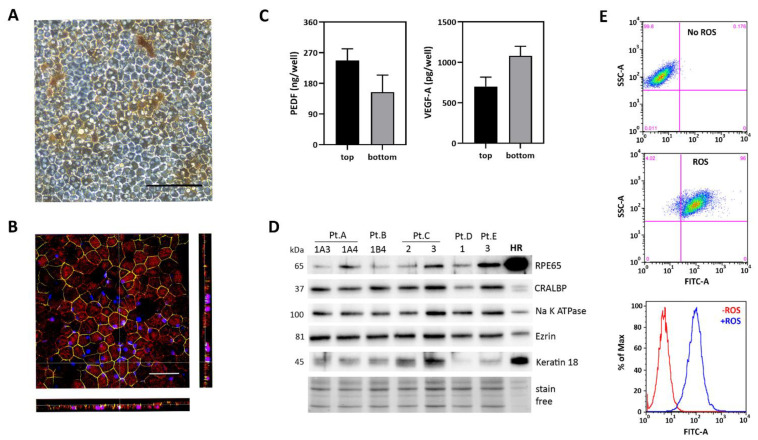
iPSC-RPE derived after reprogramming conjunctival cells obtained from patients with AMD. (**A**) Phase microscopy image showing that confluent iPSC-RPE lines form a monolayer with cobblestone appearance and have pigmentation. Scale bar = 100 µm. (**B**) Confocal microscopy image of iPSC-RPE cultured on a transwell insert. En face views of the RPE monolayer shown as maximum intensity projection through the z-axis. Bestrophin (red) is expressed on the basal surface. ZO-1 (green) marks cell borders. Nuclei are stained with DAPI (blue). Scale bar = 40 µm. (**C**) Results from ELISA analysis of pigment epithelium-derived factor (PEDF) and vascular endothelial growth factor A (VEGF-A) content measured in apical (top) and basal (bottom) media from iPSC-RPE (*n* = 5) grown on transwells. Mean ± SEM. (**D**) iPSC-RPE cultures express prototypic RPE proteins as demonstrated on Western immunoblots. Molecular mass for each protein is shown on the left. HR is a homogenate of RPE tissue from a human donor. Stain-free image is loading control. (**E**) Representative data from FACS analysis measuring the phagocytosis of FITC-labeled OS by RPE. Dot plots and histograms for cells without and with the addition of OS are shown.

Our group and others have demonstrated that iPSC-RPE lines recapitulate cardinal characteristics of native RPE [15,16,23,24,25]. Confluent patient-specific iPSC-RPE exhibited a pigmented cobblestone appearance (Figure 2A). Confocal immunofluorescent imaging of cells grown on transwells showed they attain correct polarization and form tight junctions (Figure 2B). Furthermore, these cells secrete pigment epithelium-derived factor (PEDF) preferentially to the apical side of the monolayer (1.5-fold higher) and vascular endothelial growth factor-A (VEGF-A) preferentially to the basolateral side (1.5-fold higher) (Figure 2C). Results from Western immunoblotting show the iPSC-RPE lines express proteins associated with RPE (RPE65, cellular retinaldehyde binding protein CRALBP), polarity (Na, K ATPase, Ezrin), and epithelial cells (Keratin 18) (Figure 2D). Cultures of patient-specific iPSC-RPE are also functionally similar to native RPE in vivo, as they effectively phagocytose photoreceptor outer segments (Figure 2E). Flow cytometry analysis of cells after 16 h incubation with outer segments showed that 93 ± 3% (mean ± SEM) of the cells had internalized outer segments in three different lines from two donors (data not shown).

### 2.3. Mt Testing of iPSC-RPE from Individual AMD Patients

**Figure 3:**
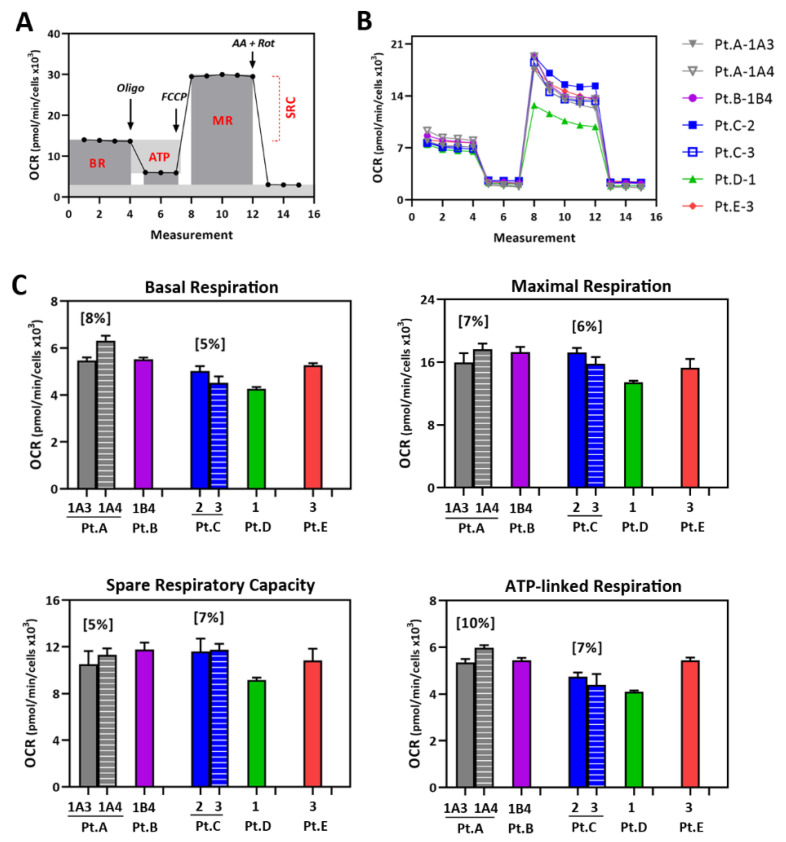
Testing mitochondrial function in iPSC-RPE from patients with AMD. (**A**) Example trace associated with Cell Mito Stress Test (CMST). Analysis of oxygen consumption rate (OCR) following injections of oligomycin (oligo), FCCP, and antimycin A + rotenone (AA + Rot) to perturb mitochondrial function. Calculation of the basal respiration (BR), maximal respiration (MR), spare respiratory capacity (SRC), and ATP-linked respiration (ATP) is shown. (**B**) Traces from CMST of OCR for patient-specific iPSC-RPE (five patients, seven lines). (**C**) Parameters of mitochondrial function were calculated from data shown in (**B**). Mean ± SEM. Numbers in brackets indicate coefficient of variation (CV) of OCR for two cell lines from the same patient.

Mt function of patient-specific iPSC-RPE was measured using an XFe96 Extracellular Flux Analyzer to perform the Cell Mito Stress Test assays. Figure 3A shows a description of the assay. This test measures oxygen consumption rate (OCR), an indicator of mt respiration. Sequential injections of mt stressors (oligomycin, FCCP, and antimycin A/rotenone) allows for the calculation of basal respiration (BR), maximal respiration (MR), spare respiratory capacity (SRC), and ATP-linked mt respiration (ATP). Figure 3B shows the OCR traces for the iPSC-RPE lines derived from the AMD patients, which were used to calculate the parameters of mt function for each cell line (Figure 3C). Multiple cell lines were generated from patients A and C. We compared their mt function and found that there was ≈8% variance in mt parameters when comparing the lines from patients A and C (Figure 3C). This variation between lines derived from the same donor was similar to results in a previous study with iPSC-RPE lines derived from multiple eye bank donors with AMD [24], where we measured ≈10 ± 2% (mean ± SEM) variance in BR and ATP, and ≈13 ± 2% variance in MR (Figure S1). In this study, data from the duplicate lines from the same patient were averaged in subsequent experiments.

### 2.4. Testing Compounds That Target Mt Activity and Homeostasis in AMD Donor iPSC-RPE

**Figure 4:**
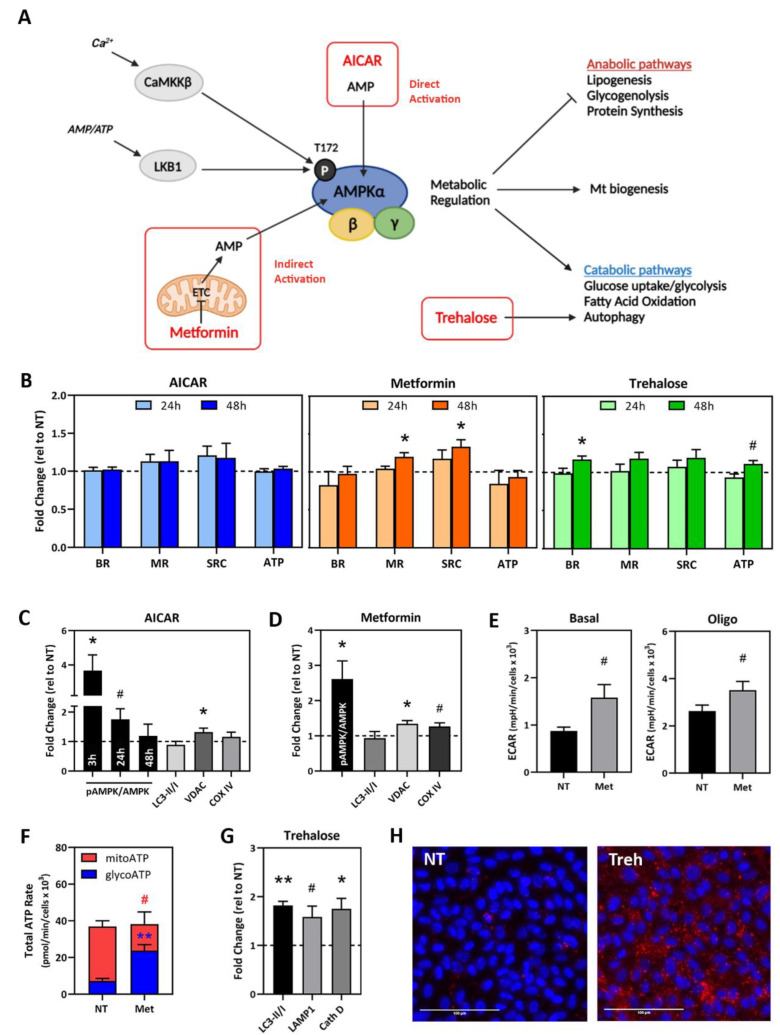
Characterization of drug treatment effect using AMD donor iPSC-RPE cells. (**A**) Schematic of AMPK activation and regulation of metabolism. (**B**) iPSC-RPE cells (*n* = 4 donors) were treated with compounds (AICAR, Metformin, or trehalose) for 24 or 48 h. Mitochondrial function was evaluated using an XFe96 Extracellular Flux Analyzer. Calculated fold change values of mt function (compared to untreated controls) for the four lines are shown. BR = basal respiration; MR = maximal respiration; SRC = spare respiratory capacity; ATP = ATP-linked respiration. (**C**,**D**) Protein content in lysates from iPSC-RPE cells (*n* = 4 donors) following incubation with 500 µM AICAR (**C**) or 2 mM Metformin (**D**). Calculated fold change values are shown (no treatment = 1, dashed line). (**E**) ECAR was measured during CMST assays (*n* = 4 donors). (**F**) ATP Rate Assay was performed on iPSC-RPE (*n* = 4) after Metformin treatment for 48 h. (**G**) iPSC-RPE (*n* = 4 donors) were treated with 100 mM trehalose for 48 h. Content of autophagy-related proteins was determined in treated cells relative to untreated cells (dashed line). LC3-II/I, Lysosomal-Associated Membrane protein 1 (LAMP1), Cathepsin D (Cath D). (**H**) Maximal projection of z-stack images of LysoTracker™ staining (red) labeling lysosomes in untreated (NT) and trehalose treated (Treh) iPSC-RPE cells. Nuclei are stained with DAPI (blue). Scale bar = 100 µm. Data in (**A**–**G**) are mean ± SEM. One-sample *t*-tests were used to compare treatment to no treatment in (**C**,**D**,**G**). Unpaired *t*-tests were used to compare treatment to NT in (**A**,**E**,**F**). ** *p* < 0.01, * *p* < 0.01, # *p* < 0.1.

For this study, we selected three compounds (AICAR, Metformin, trehalose) that target mt function and homeostasis. AICAR and Metformin are pharmacological activators of 5′ adenosine monophosphate protein kinase (AMPK), which is a key regulator of energy metabolism. AMPK directly binds to AMP, ADP, or ATP, allowing the detection of energy levels in the cell. The activation of AMPK promotes downstream energy producing pathways and inhibits energy-consuming pathways [26,27] (Figure 4A). Trehalose increases autophagy, which is a process used to eliminate damaged mitochondria, by inducing lysosomal expansion.

Preliminary experiments to confirm the effect of these drug treatments were performed using iPSC-RPE lines previously derived in our laboratory from 10 individual eye bank donors with AMD (1 line/donor) [23,24]; see Table S1 for individual donor demographics and their use in Figure 4. Optimal doses were chosen from published studies using RPE cells [28,29,30,31]. Optimal timing of short-term treatment was determined by performing the CMST (Cell Mito Stress Test) assay in donor iPSC-RPE cells following 24 or 48 h of drug treatment (Figure 4B). Compared to no treatment, Metformin treatment (48 h) significantly increased MR (*p* = 0.03) and SRC (*p* = 0.03). Trehalose treatment (48 h) significantly increased BR (*p* = 0.03). Small increases in MR and SRC were observed with AICAR; however, it did not reach statistical significance. From these data, we selected 48 h treatment for subsequent experiments.

Additional characterization included monitoring changes in the content of specific proteins following treatment with this panel of drugs. Representative Western blot images are found in Figure S2. AICAR and metformin activate AMPK via the phosphorylation of Threonine 172 [20]. We found that while metformin treatment for 48 h significantly increased the pAMPK/AMPK ratio (*p* = 0.05), AICAR did not (Figure 4C,D). Since AICAR is an AMP analog, we tested for an earlier response and found the ratio of pAMPK/AMPK was elevated 3.7-fold at 3 h (*p* = 0.03) with a gradual decrease to baseline by 48 h (Figure 4C).

The activation of AMPK upregulates mt biogenesis. To assess the effect of AICAR or metformin treatment, we measured the content of voltage-dependent anion channel (VDAC) and cytochrome c oxidase subunit IV (COX IV). These proteins are located on the outer and inner mt membranes, respectively, and they are used to estimate mt content. We found that AICAR increased VDAC content (*p* = 0.05; Figure 4C), and Metformin increased both VDAC (*p* = 0.03) and COX IV (*p* = 0.07) content (Figure 4D). We also observed that metformin causes a shift in metabolism. Using the extracellular acidification rate (ECAR) values from the CMST assays, Metformin treatment caused 1.3–1.5 fold increases in the basal level of ECAR (*p* = 0.08) and ECAR after oligomycin injection (*p* = 0.07) (Figure 4E). These results suggest that cells exposed to metformin had elevated glycolysis. This change was not observed after AICAR treatment (Figure S3), suggesting different mechanisms of action for these drugs. Using the ATP Rate Assay to confirm ECAR results, we found that Metformin treatment causes RPE cells to produce more ATP from glycolysis (*p* < 0.01) and less ATP from mitochondria (*p* = 0.08), although the total ATP rate did not change (Figure 4F).

Since AMPK also regulates catabolic pathways including autophagy, we measured the ratio of microtubule-associated protein 1 light chain 3 bands (LC3-II/I) to monitor autophagosome content. We found there was no change in LC3-II/I after AICAR or metformin treatment (Figure 4B,C). We also tested the effect of trehalose, which is a compound known to increase autophagy. We observed increases in the content of the autophagy marker, LC3 (*p* < 0.01), and lysosomal markers, LAMP1 (*p* = 0.06) and Cathepsin D (*p* = 0.03) after trehalose treatment compared to no treatment (Figure 4G). Lysosomal labeling, using the fluorescent marker LysoTracker™, was also increased in trehalose-treated cells compared to untreated cells (Figure 4H). These results are consistent with trehalose inducing the expansion of the lysosomal compartment, resulting in an increase in overall autophagy.

### 2.5. Drug Testing in Patient-Specific iPSC-RPE Using AMPK Activators

**Figure 5:**
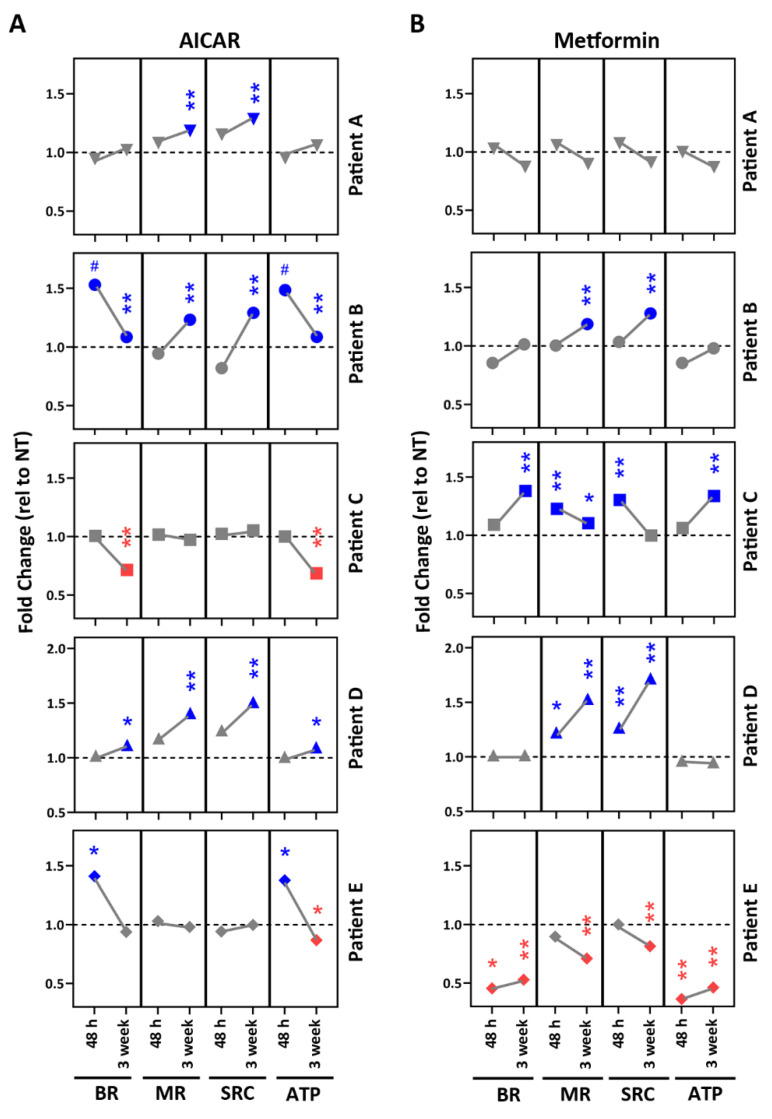
Testing AMPK activators using patient-specific iPSC-RPE. In two separate experiments, iPSC-RPE cells from patients with AMD (*n* = 5) were treated with (**A**) AICAR or (**B**) Metformin for 48 h or 3 times per week for 3 weeks. Following treatment, parameters of mt function were calculated from OCR measured using an XFe96 Extracellular Flux Analyzer. OCR data were normalized to cell count per well. Graphs show fold change relative to each donor’s no-treatment control (dashed line). Blue data points indicate response from cells that exhibited improved mt function. Red data points indicate response from cells that exhibited decreased mt function. BR = basal respiration; MR = maximal respiration; SRC = spare respiratory capacity; ATP = ATP-linked respiration. * *p* < 0.05, ** *p* < 0.01, # *p* < 0.1 determined by unpaired *t*-tests of raw OCR values (average no treat OCR vs. average treatment OCR).

After confirming exposure to AICAR and Metformin activated AMPK and altered metabolism in a cohort of iPSC-RPE lines, we next examined the response of our patient-derived iPSC-RPE lines to treatments in two separate experiments (Figure 5). In the first experiment, the patient-specific iPSC-RPE was subjected to an acute drug exposure (48 h), and in the second experiment, a chronic drug exposure (3-week) was used to more closely mimic a sustained therapeutic regimen for patients.

Figure 5 compares the results of short- (48 h) and long-term (3-week) treatment of the iPSC-RPE lines generated from five patients to the AMPK activators, AICAR (A), and metformin (B). The graphical summary emphasizes both the magnitude and pattern of changes in mt metabolism. Adverse responses are designated by the red symbols. Beneficial effects are shown in blue. Overall, there was high variability in how each cell line responded to individual drugs and which mt parameters were affected. In general, the 3-week regimen elicited a more robust response. Two patient lines (B and D) exhibited positive effects to both AMPK activators. The other three patient lines (A,C,E) exhibited opposite effects to the two drugs. Of note, Patient E had a negative response to Metformin.

### 2.6. Drug Testing in Patient-Specific iPSC-RPE Using an Autophagy Inducer

**Figure 6:**
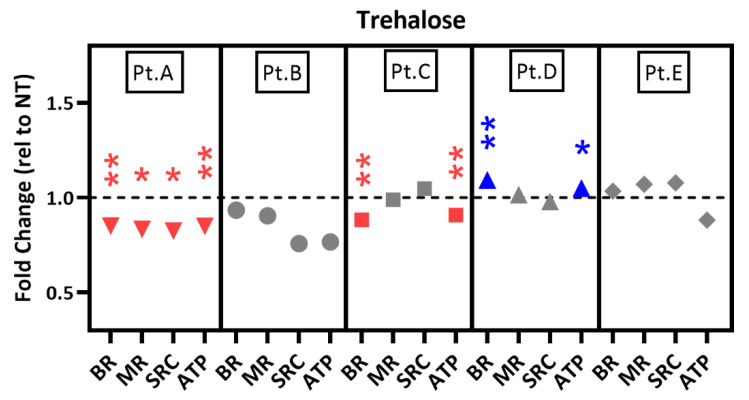
Testing an autophagy inducer using AMD patient-specific iPSC-RPE. iPSC-RPE cells from patients with AMD (*n* = 4) were treated with trehalose for 48 h. Following treatment, parameters of mt function were calculated from OCR measured using an XFe96 Extracellular Flux Analyzer. OCR data were normalized to cell count per well. Graphs show the fold change relative to each donor’s no-treatment control (dashed line). Blue data points indicate response from cells that exhibited improved mt function. Red data points indicate response from cells that exhibited decreased mt function. BR = basal respiration; MR = maximal respiration; SRC = spare respiratory capacity; ATP = ATP-linked respiration. * *p* < 0.05, ** *p* < 0.01 determined by unpaired *t*-tests of raw OCR values (average no treat OCR vs. average treatment OCR).

Having confirmed the effect of trehalose in iPSC-RPE cells (Figure 4), we tested its short-term effect on mt function in five patient iPSC-RPE lines compared with untreated cells (Figure 6). As seen with AICAR and Metformin, the response to trehalose was variable and patient-specific. Patients B and E were unresponsive. Trehalose had a negative effect on mitochondria in cells from Patients A and C. Only Patient D responded positively; BR and ATP production were slightly increased. Long-term exposure (3-week) at the dose used (100 mM) resulted in cell death, so we were not able to compare short- and long-term exposure with this drug.

## 3. Discussion

In this proof-of-concept study, we generated iPSC-RPE derived from five AMD patients graded for disease severity and genotyped for two SNPs (CFH Y402H and ARMS2 A69S) associated with the highest risk of developing AMD. These SNPs were abundantly represented in our patient sample with 100% harboring the high-risk allele for ARMS2 and 80% harboring the high-risk allele for CFH. The iPSC-RPE lines from these patients were used in a small-scale drug-screening platform designed to evaluate the efficacy of compounds that target key processes in mt homeostasis. Our results demonstrate the feasibility of using iPSC-RPE from AMD patients to help develop a personalized drug treatment regime and provide a roadmap for future clinic management of this disease.

iPSC-RPE lines provide a number of advantages, including the ability to generate iPSC-RPE from a variety of somatic cells. Importantly, the cell source used for reprogramming does not alter the RPE phenotype or functional characteristics, as shown by similarities reported between iPSC-RPE derived from primary RPE, skin fibroblasts, or cornea [32,33]. iPSC-RPE can also provide the opportunity for modeling disease on a patient-by-patient basis. This was shown in previous studies where patient-specific iPSC-RPE lines were used to investigate both monogenetic retinal diseases [34,35,36,37,38] as well as disease arising from both genetic and environmental factors, including AMD [15,24,25,39,40].

Another advantage is the ability to expand iPSC-RPE extensively in culture, making preclinical drug testing possible. Only a few groups have begun to explore using iPSC-RPE to test potential therapeutic drug candidates for AMD [15,16,41]. Results show that nicotinamide, known for its antioxidant and anti-inflammatory properties, ameliorated the AMD disease phenotype [15]. Curcumin and ciclopirox olamine, which are bioactive FDA-approved drugs, both protected iPSC-RPE from oxidative stress [16,41]. The efficacy of these compounds suggests they could target retinal oxidative stress and inflammation, which are conditions that are associated with AMD onset and progression [42,43]. Taken together, the results in these published papers and the current study support the use of iPSC-RPE to investigate therapeutics to treat the underlying mechanism causing the AMD phenotype.

Our current study used a comprehensive assay of mt function to evaluate the efficacy of multiple drugs with the potential to improve mt health. The selection of drugs was based on experimental findings of specific defects associated with AMD. For example, energy metabolism pathways are dysfunctional in RPE from AMD donors [8,9,44]; therefore, regulators of these pathways, such as AMPK, may be ideal targets to treat AMD. Initial testing demonstrated that AICAR and Metformin activate AMPK and promote mt biogenesis (Figure 4) possibly through PGC-1α activation [26]. AICAR, an analog of AMP, is a direct and rapid activator of AMPK. Although the exact mechanism is unknown, metformin is a purported indirect activator of AMPK that acts by changing the AMP/ATP ratio through the inhibition of Complex I of the electron transport chain [45]. Metformin also improves mt function by promoting mt fission [46]. In vivo effects of Metformin include protecting RPE in mouse models of retinal degeneration [47] and decreasing the odds ratio for developing AMD in diabetic patients [48,49].

Improvements shown in RPE mt function after in vitro AICAR and Metformin treatment suggest that these drugs are able to overcome or repair the underlying cellular defect in selected patient’s cells. The drug treatments may enhance mt remodeling to induce the formation of new organelles, leading to an overall improvement in mt function. It is important to note that relatively small improvements in mt function may provide sufficient resilience against stress, aging, or inflammation in the RPE to prevent cell loss and retain visual function.

Dysfunctional autophagy is another defect reported in RPE from AMD donors that could have negative consequences on mt function by allowing defective mitochondria to accumulate [9]. Incubation of iPSC-RPE with the sugar trehalose upregulated autophagy via lysosomal expansion, as demonstrated by the increase in LC3-II and lysosomal markers (Figure 4). However, under our experimental conditions, acute treatment with trehalose did not improve or had a negative effect on mt function in most patient cells. While we did not investigate the mechanism responsible for the negative response, it is possible that the over-activation of autophagy could reduce mt function. In previous testing with the alternative autophagy inducer rapamycin, we observed improved mt function in primary RPE cultures from AMD donors [13]. Rapamycin upregulates autophagy by inhibiting mTOR, while trehalose is an mTOR-independent inducer of lysosomal expansion [50]. Thus, the difference in response to trehalose and rapamycin could be due to these drugs affecting different pathways, or that there is no lysosomal defect in this population of iPSC-RPE.

Consistent with the previous drug studies [13,15,16,41], we observed variability in drug response across patient cell lines (Figure 5 and Figure 6). The unique patterns of drug response that we observed may provide clues to specific metabolic defect(s) causing mt dysfunction in AMD patients. A lack of response may indicate that the compounds tested do not address the metabolic defects. For example, Patients B and D responded positively (increased mt function) to both AICAR and Metformin. These results support the idea that these patients may have defects in the AMPK pathway. Patients A, C, and D had opposite responses to Metformin and AICAR, suggesting specific differences in the site of defect of the energy-sensing pathway. The other important information from the drug screening was the identification of drugs that are detrimental to mt function, as seen for Patient E for metformin treatment. This observation is clinically relevant, since Metformin is widely used for treating diabetes, cancer, and more recently, aging [51]. Thus, screening patient-derived cells for negative or positive effects could provide a way to avoid detrimental side effects of specific drugs for individual patients.

In this proof-of-concept study, we avoided testing unverified candidate molecules in proprietary chemical libraries and instead selected drugs that have been approved by regulatory authorities for treating different diseases. Our rationale was that repurposing drugs with known properties (e.g., toxicity profile, dose, side effects, and established delivery modality) could significantly reduce the timescale between the identification of potentially efficacious compounds and clinical testing [52]. The three drugs tested in the current study are being used in clinical trials or are prescribed treatments, so their safety has been established. AICAR is in a phase II pilot study for a genetic disorder called Lesch-Nyhan Syndrome (NCT00004314). While Metformin is prescribed for conditions, such as diabetes, cancer, and aging, there is one phase II clinical trial for oral Metformin treatment for slowing the progression of dry AMD (NCT02684578). Trehalose, applied topically in the form of eye drops, is in clinical trials for dry eye (NCT03569202, NCT01742884, NCT04803240). The next step in finding the most efficacious treatments for AMD involves expanding the panel of drugs and including cell lines from additional patients. It is also important to consider the patient’s genetic background. Screening for SNPs or other genetic markers associated with high risk for AMD may aid in choosing the right population for testing AMD therapeutics. For example, our previous study found that iPSC-RPE derived from CFH high-risk donors had decreased mt function compared to CFH low-risk iPSC-RPE [53], suggesting that this patient population would benefit from mt-targeted drug therapy. The goal is to extrapolate the information gained from in vitro testing of patient-specific iPSC-RPE to in vivo treatment of patients with AMD.

Despite this study having a small sample size of patients and testing a limited number of drugs, we have demonstrated the potential for individualized drug treatments. Future directions include increasing the scale of iPSC derivation and cell differentiation for entire patient populations and increasing the number of drugs tested. Recent improvements in defined cell culture systems, efficient and reliable reprogramming reagents, as well as more reproducible differentiation protocols, have increased the utility of iPSC technology in high-throughput screening. However, expanding this technology to whole patient cohorts will require the application of automation systems to reduce the manpower, timescale, and cost associated with deriving lines from multiple patients. This process is underway in our group as well as in other laboratories [54,55,56].

While future large-scale drug screening in iPSC-RPE from AMD patients should include the three drugs tested in this study, it should also include multiple drugs that target pathways demonstrated to maintain and/or restore mt function. These pathways include mt biogenesis; mt dynamics, including fusion and fission; and mt quality control through mitophagy and proteostasis [57]. It may be beneficial to use several drugs that influence the same pathway, since each drug has a different mechanism of action. For example, when testing drugs that stimulate mt biogenesis, it would be useful to not only include AICAR and Metformin in the drug panel but resveratrol and NAD+ precursors as well. Although all of these compounds may increase mt biogenesis, each targets a different part of the pathway. A patient may have a defect in one or more parts of the mt biogenesis pathway, so including multiple drugs would allow for easier identification of treatments.

## 4. Materials and Methods

### 4.1. Conjunctiva Biopsies from AMD Patients

The identification of study participants and tissue collection was conducted with approval from the University of Minnesota (UMN) Institutional Review Board (Study number 00000851, date of approval 4 December 2020). Conjunctival biopsies were collected from fully consented UMN study participants attending the Ophthalmology clinic at the University of Minnesota. Evaluation of the patient’s stage of AMD was determined by a Board-Certified Ophthalmologist (S.R.M.) from fundus images using the criteria established by the Age-related Eye Disease Study (AREDS) [53]. A 2–3 mm piece of conjunctiva was collected from one eye of study participants and immersed in KGM-2 medium (3 mL) (Lonza Bioscience, Morrisville, NC, USA; cc-3101, cc-4152) and stored at 4 °C before transport to the Stem Cell Institute. Processing of the conjunctival tissue for adherent culture was performed within 16 h of the biopsy collection. To initiate adherent culture of primary conjunctival cells, the biopsy tissue was put in the center of a 6 cm plate and cut into 8–10 small pieces. Pieces of the conjunctival tissue were dispersed and allowed to partially air dry before being covered with KGM-2 (30 µL) keratinocyte growth medium and connected by a media bridge to a 3 mL reservoir of media before incubation overnight at 37 °C, 5% CO_2_. KGM-2 media (1 mL) was added to the media reservoir every 2 days for 7 days. After 7 days, the medium was replaced with fresh KGM-CD (5 mL) medium and replaced every 2 days for another 7 days. Then, the cells were harvested using TrypLE™ Select (Thermo Fisher Scientific, Waltham, MA, USA; 12563-011).

### 4.2. Culturing iPSC-RPE Cells

The derivation of iPSC lines from primary human conjunctival cells, differentiation of iPSC to RPE, and expansion of iPSC-RPE has been described in our previous publication [24]. iPSC-RPE cells from passage 3 were used for the characterization and functional assays. The iPSC-RPE lines used in this study are listed in Table 1.

### 4.3. Genotyping

Genomic DNA was extracted from a pellet of conjunctival cells using QIAamp® DNA Micro kit (Qiagen; Germantown, MD, USA; 56304). DNA was quantified using a Quant-iT PicoGreen dsDNA assay kit (Thermo Fisher Scientific; Waltham, MA, USA; P7589). Samples were genotyped for the Complement Factor H (CFH) variant Y402H using allele-specific primers designed for the single nucleotide polymorphism (SNP) rs1061170. CFH-Y402H-F: TGAGGGTTTCTTCTTGAAAATCA, CFH-Y402H-R: CCATTGGTAAAACAAGGTGACA, and genotyped for Age-related maculopathy susceptibility 2 (ARMS2) variant A69S using primers designed for SNP rs10490924. ARMS2-A69S-F: TCCTGGCTGAGTGAGATGG, ARMS2-A69S-R: GGCATGTAGCAGGTGCATT. The PCR product purified with Gel PCR DNA fragments extraction kit (IBI Scientific; Dubuque, IA, USA) was submitted for classic Sanger sequencing (UMN Genomics Core). Base calling was manually inspected using Sequence Scanner 2 software version 2.0 (Applied Biosystems; Waltham, MA, USA).

### 4.4. Immunofluorescence

Immunofluorescence was performed on iPSC-RPE cells as described [24]. The antibodies used in this study are listed in Table S2.

To image lysosomes, iPSC-RPE cells (4 × 10^4^ cells/well) were grown in Matrigel-coated 96-well black/clear bottom plates, treated with trehalose (100 mM) for 48 h, and incubated with LysoTracker™ Red DND-99 (50nM; Fisher Scientific; Hampton, NH, USA) for 30 min. Hoechst 33342 was added to stain the nucleus. Cells were imaged using Cytation1 imager (BioTek; Winooski, VT, USA). 

### 4.5. Enzyme-Linked Immunosorbent Assay (ELISA)

RPE cells were seeded at a density of 4 × 10^5^ cells/well in 6.5 mm diameter polyester inserts (0.4 µm pores; Corning, Inc., Corning, NY, USA) coated with Matrigel^®^. ELISA for vascular endothelial growth factor A (VEGF-A) (Thermo Fisher Scientific; Waltham, MA, USA; BMS277/2) and pigment epithelium-derived factor (PEDF) (R & D Systems; Minneapolis, MN, USA; DY1177-05) were performed as described [53]. Growth factor concentrations were derived from standard curves and corrected for chamber volume differences.

### 4.6. Western Blotting

Cell pellets were collected and lysed in RIPA buffer (Sigma-Aldrich, St. Louis, MO, USA). Protein concentrations were determined with BCA assay (Thermo Fisher Scientific; Waltham, MA, USA) using albumin as the standard. Western blots were performed as described [24]. Membranes were incubated overnight with primary antibodies (see Table S2). Images of immune reactions were taken using a BioRad ChemiDoc XRS.

### 4.7. Phagocytosis of Outer Segments (OS)

Bovine outer segments (InVision BioResources; Seattle, WA, USA) were labeled with Fluorescein-5-Isothiocyanate Isomer I (FITC) (Thermo Fisher Scientific; Waltham, MA, USA). Labeled OS were pelleted, washed, and added to confluent iPSC-RPE cultures at a concentration of 40 OS/RPE cell. After 16 h of incubation, cells were washed with PBS, dissociated with trypsin, and collected in PBS for analysis. Flow cytometry analysis was performed using LSRII H1010 (BD Biosciences; Franklin Lakes, NJ, USA), and the data were analyzed with FlowJo software version 9.

### 4.8. Measuring RPE Mt Function Using the Cell Mito Stress Test (CMST)

Mt function in treated and untreated iPSC-RPE was measured using the XFe96 Extracellular Flux Analyzer (Agilent Technologies; Santa Clara, CA, USA) and the Cell Mito Stress Test (CMST) assay. Briefly, cells were plated in MEM alpha medium (Gibco) containing 1% FBS (Atlanta Biologicals; Flowery Branch, GA, USA), pen/strep (50 U/mL/50 µg/mL), sodium pyruvate (1 mM), GlutaMAX™ (1X), non-essential amino acids (1X), N1 supplement (1X), taurine (0.25 mg/mL), hydrocortisone (0.02 µg/mL), tri-iodothyronine (0.013 µg/L), and seeded (4 × 10^4^ cells/well) on XF96 plates coated with Matrigel^®^. For each patient iPSC-RPE line, five technical replicates were assayed for each condition. The following day, RPE cells were treated with 500 µM AICAR (Sigma-Aldrich; St. Louis, MO, USA), 100 mM trehalose (Sigma-Aldrich; St. Louis, MO, USA), or 2 mM Metformin (Cayman Chemical; Ann Arbor, MI, USA), and after 48 h, CMST assays were run. For long-term treatment, cells were plated in Matrigel®-coated 12-well plates and then treated three times per week with one of the drugs. Following three weeks of treatment, cells were dissociated from the 12-well plates, seeded into XF96 plates, and treated with the same drug for 48 h prior to performing the CMST assay.

The CMST assay protocol was performed according to the manufacturer’s instructions (Agilent Technologies; Santa Clara, CA, USA) and our previous analyses [8,13,24]. The oxygen consumption rate (OCR) was detected before and after the sequential addition of oligomycin (2 μM), FCCP (1 μM), and finally rotenone (1 μM) and antimycin A (1 μM). The resultant changes in OCR allowed the calculation of basal respiration, ATP-linked respiration, spare respiratory capacity, and maximal respiration. Hoechst 33342 dye was added in the third injection to enable post assay cell count at 10X magnification using a Cytation1 imager (BioTek; Winooski, VT, USA). Data processing used Wave software version 2.0 (Agilent Technologies) normalizing OCR to cell count.

### 4.9. Measuring ATP Production Rates Using Real-Time ATP Rate Assay

Total ATP production rates in treated and untreated iPSC-RPE was measured using the XFe96 Extracellular Flux Analyzer (Agilent Technologies) and the ATP Rate assay using the same conditions as the CMST assay (described above). The OCR and Extracellular Acidification Rate (ECAR) were detected after a serial injection of oligomycin and antimycin A/rotenone to allow for the calculation of mitochondrial and glycolytic ATP production rates. Data were normalized to cell count.

### 4.10. Statistical Analysis

Treatment data were normalized to the no-treatment condition for each donor (fold change relative to no treatment). For Western blots, one-sample *t*-tests were performed on log-transformed fold change values. For CMST and ATP-Rate assays, unpaired *t*-tests were performed on the five technical replicates of OCR values comparing no treatment values to each treatment values. Analyses were performed using the statistical software in GraphPad Prism 9. Probability ≤0.05 was considered statistically significant.

## 5. Conclusions

In this proof-of-concept study, we provide a roadmap for drug testing using patient-derived iPSC-RPE to identify treatments that are either beneficial or detrimental to mt RPE function. Since there are multiple cellular defects that can manifest the dry AMD phenotype, no single drug will be suitable to treat all patients [18]. A more targeted patient-specific approach is needed to find specific drugs that will restore or improve RPE mt health for individual patients with dry AMD. While our focus is on finding a treatment for dry AMD, this approach is applicable for many other diseases without approved treatments or as a prescreening tool to identify the best treatment for individual patients.

## Figures and Tables

**Table 1:** Patient iPSC-RPE lines and donor demographics.

iPSC-RPE Line ID	Age ^a^/Gender ^b^	Disease Stage ^c^	CFH ^d^/ARMS2 ^e^ Genotype	Figures Using Data from Specific Lines
Pt.A-1A3 ^+^	84/F	AREDS3	CT/GT	2C-E, 3B-C, 5, 6
Pt.A-1A4 ^+^	84/F	AREDS3	CT/GT	2C-D, 3B-C, 5, 6
Pt.B-1B4	76/F	AREDS4	CC/TT	2B-D, 3B-C, 5, 6
Pt.C-2 ^#^	65/M	AREDS3	CC/TT	2C-D, 3B-C, 5, 6
Pt.C-3 ^#^	65/M	AREDS3	CC/TT	2A,C-D, 3B-C, 5, 6
Pt.D-1	76/F	AREDS3	CC/GT	2C-D, 3B-C, 5, 6
Pt.E-3	63/F	AREDS2	TT/GT	2C-D, 3B-C, 5, 6

^+^ Lines derived from the same patient. ^#^ Lines derived from the same patient. ^a^ Age of patient, in years, from whose conjunctival cells were used to generate iPSC-RPE. ^b^ Gender of patient. F = female, M = male. ^c^ AREDS System used to evaluate the stage of AMD in biopsy donor. AREDS category 2 = early AMD, AREDS category 3 = intermediate AMD, AREDS category 4 = advanced-stage AMD [23]. ^d^ Complement Factor H (CFH) genotype for rs1061170; low risk = TT, high risk = CT and CC. ^e^ Age-related maculopathy susceptibility 2 (ARMS2) genotype for rs10490924; low risk = GG, high risk = GT and TT.

## Data Availability

Data used to support the findings of this study are contained within this article and supplementary material.

## Supplementary Materials

The following are available online at https://www.mdpi.com/article/10.3390/ph15010062/s1, Figure S1: Determining the degree of concordance between iPSC-RPE lines generated from the same donor, Figure S2: Representative Western blot images from data shown in Figure 4, Figure S3: AICAR does not cause a shift in metabolism, Table S1: AMD donor iPSC-RPE demographics, Table S2: List of antibodies used in this study.
