# Supplementary material for: Testing Mitochondrial-Targeted Drugs in iPSC-RPE from Patients with Age-Related Macular Degeneration
Source: Pharmaceuticals (Basel). 2022 Jan 4;15(1):62. doi: 10.3390/ph15010062 (PMC8781759; doi:10.3390/ph15010062)
Supplement: Supplementary file 1 [file pharmaceuticals-15-00062-s001.zip › pharmaceuticals-1517150-supplementary.pdf]

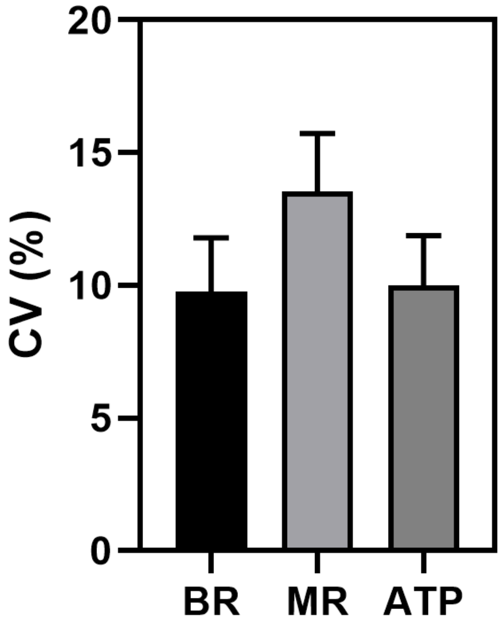

Figure S1. *Determining the degree of concordance between iPSC-RPE lines generated from the same donor.* To compare multiple lines from the same donor (n=15 total donors), coefficient of variance (CV= (stdev/mean)*100) was calculated from the results of mt parameters measured in the CMST assay. Bar graphs are mean ± SEM.

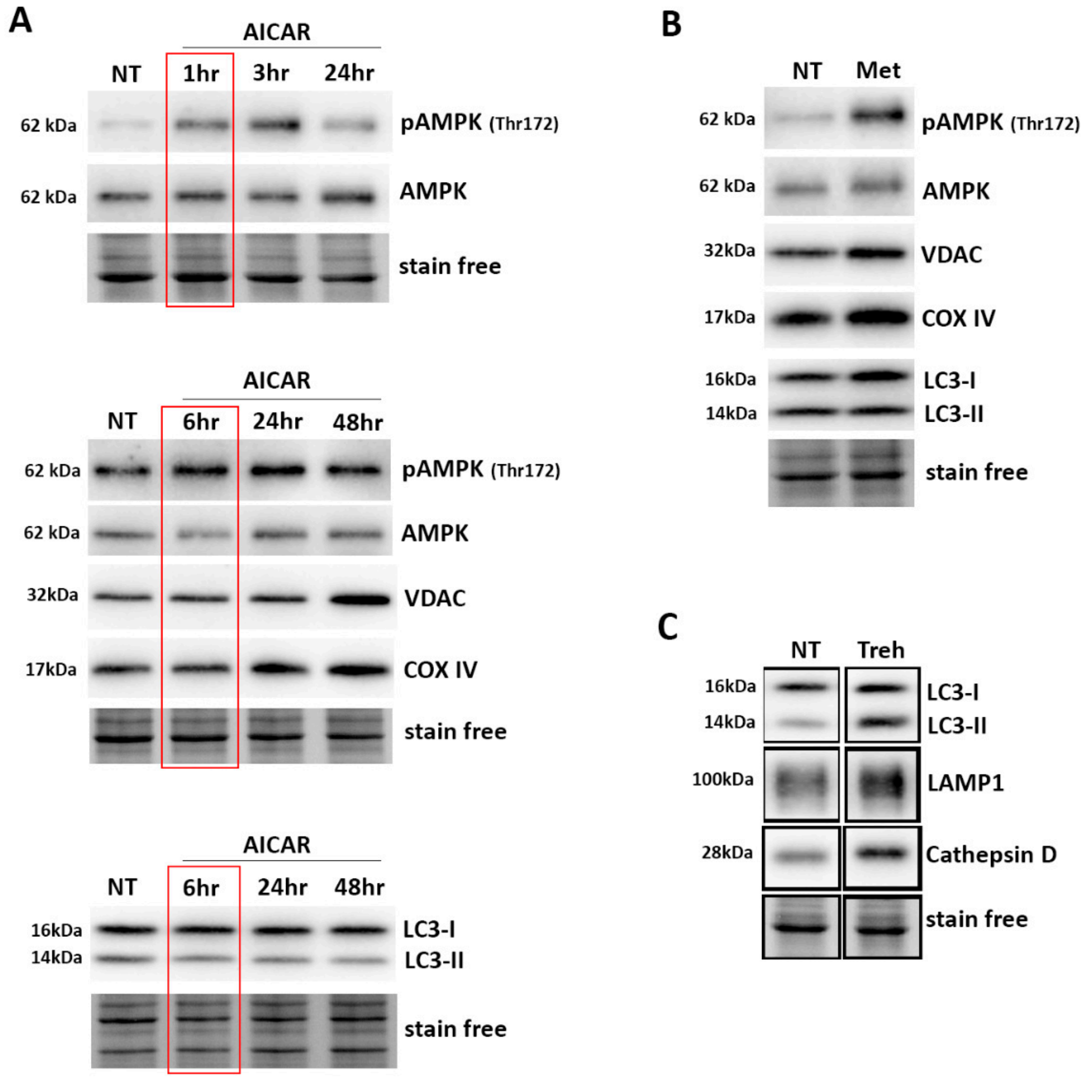

Figure S2. *Representative Western blot images from data shown in Figure 4.* Images of Western blots used to analyze protein content after AICAR (A), Metformin (Met) (B), or Trehalose (Treh) (C) treatment. NT = no treatment. Molecular mass for each protein is shown on the left. Stain free image below blot was used to normalize protein load. In Panel A, bands within the red rectangles were not used in this study.

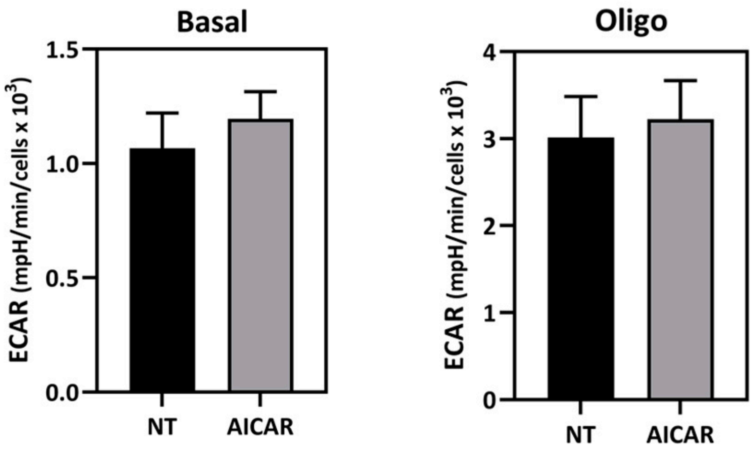

Figure S3. *AICAR does not cause a shift in metabolism.* AMD iPSC-RPE (n=4) were plated in a 96 well plate, incubated overnight and treated with AICAR for 48hr. After performing a CMST assay, ECAR values were compared between no treatment and AICAR treatment. Bar graphs are mean ± SEM.

Table S1. AMD donor iPSC-RPE lines demographics.

iPSC-RPE line	Age[a]/Gender[b]	CFH[c]/ARMS2[d] genotype	Figures using data from specific line
AMD donor 1 – 1B1	79/M	CT/TT	4C,G
AMD donor 2 – 1A6	84/F	TT/TT	4B,E,F
AMD donor 3 – 1C1	80/F	CT/GT	4B-H
AMD donor 4 – 3	83/F	TT/GT	4B,E
AMD donor 5 – 3	75/F	CC/GT	4C
AMD donor 6 – 1	75/F	CC/GT	4C,D,F,G
AMD donor 7 – C	64/F	CT/GT	4C
AMD donor 8 – 2	66/M	CT/TT	4B,E,F
AMD donor 9 – 3	70/F	CC/GG	4F,G
AMD donor 10 – C	80/F	TT/GT	4C

[a] Age of donor, in years, from whose conjunctival cells were used to generate iPSC-RPE.

[b] Gender of donor. F = female, M = male.

[c] Complement Factor H (CFH) genotype for rs106117; low risk = TT, high risk = CT and CC.

[d] Age-related maculopathy susceptibility 2 (ARMS2) for rs10490924; low risk = GG, high risk = GT and TT.

Table S2. Antibodies used in this study

Antigen	Host	Company	Product	Dilution	Application
RPE65	Mouse	Novus	NB100-355	1:1000	WB
CRALBP	Mouse	Novus	NB100-73492	1:1000	WB
Na,K ATPase	Rabbit	Cell Signaling	3010	1:1000	WB
Ezrin	Rabbit	Cell Signaling	3145	1:1000	WB
Keratin 18	Mouse	Cell Signaling	4548	1:1000	WB
ZO-1	Rabbit	Thermo Scientific	617300	1:50	IF
Bestrophin	Mouse	Novus	NB300-164	1:50	IF
pAMPK (Thr172)	Rabbit	Cell Signaling	2535	1:1000	WB
AMPK	Rabbit	Cell Signaling	2532	1:1000	WB
VDAC	Rabbit	Cell Signaling	4866	1:1000	WB
COX IV	Mouse	Cell Signaling	11967	1:1000	WB
LC3	Rabbit	Cell Signaling	4108	1:1000	WB
LAMP1	Rabbit	Cell Signaling	9091	1:1000	WB
Cathepsin D	Rabbit	Cell Signaling	2284	1:1000	WB
